# Supplementary material for: Anti-c-MET Fab-Grb2-Gab1 Fusion Protein-Mediated Interference of c-MET Signaling Pathway Induces Methuosis in Tumor Cells
Source: Int J Mol Sci. 2022 Oct 10;23(19):12018. doi: 10.3390/ijms231912018 (PMC9569552; doi:10.3390/ijms231912018)
Supplement: Supplementary file 1 [file ijms-23-12018-s001.zip › ijms-1902319-Supplementary Material .pdf]

Supplementary methods

1. OPAL multiplex immunohistochemistry analysis

Immunofluorescence (IF) was performed on paraffinized tissue sections using the Opal multiplex immunohistochemistry system (PerkinElmer, Waltham, MA). All the fluorophores used were Opal™ Dyes (Perkin Elmer), including Opal 520, Opal 570, Opal 620, and Opal700. The following primary antibodies were used: anti-cytokeratin (CK), anti-F4/80 (#ab111101), anti-CD11B (#ab133357), and anti-Ly6G (#ab238132) (Abcam, Cambridge, UK). The non-specific protein binding was removed by incubation with goat anti-mouse IgG (Thermo Fisher Scientific, MA, USA). The slides were incubated with primary antibodies for 1h, followed by incubation with polymer HRP-conjugated Ms+Rb secondary antibodies for 10min at room temperature. Then opal fluorophores were pipetted onto each slide for a 10min incubation at room temperature. Finally, nuclei were counterstained with DAPI.

Supplementary **Table S1**.Amino acid sequence of Gab1-MBD16 peptide and Grb2-SH2 peptide.

Peptide	Amino Acid Sequence
Gab1-MBD16	FGMQVPPPAHMGFRSS
Grb2-SH2	KAHPWFFGKIPRAKAEEMLNKQRHDGAFLIRESESAPGDFSLSVKFGNDVQHFKVLRDGAGKYF<br>LWVVKFNSLNELVDYHRTTSVSRNQQIFLRD
ZWB90 H1	QVQLVQSGAEVKKPGASVKVSCKASGYTFTDYYMHWVRQAPGQGLEWMGRVNPNRRGTTYN<br>QKFEGRVTMTTDTSTSTAYMELRSLRSDDTAVYYCARANWLDYWGQGTTVTVSSASTKGPSVF<br>PLAPSSKSTSGGTAILGCLVKDYFPEPVTVSWNSGALTSGVHTSPAVLQSSGLYMLASAVTVPSS<br>SLGTQTYICNVNHKPSNTKVDKKVEPKSCDKTHTPPCPAPELLGGPSGGGGSFGMQVPPPAHM<br>GFRSS
ZWB90 L1	DIQMTQSPSSLSASVGDRVTITCSVSSSVSSIYLHWYQQKPGKAPKLLIYSTSNLASGVPSRFSGS<br>GSGTDFTLTISSLQPEDFATYYCQVYSGYPLTFGGGTKVEIKRTVAAPSVAIFPPSDEQLKSGTAS<br>VVCVLNNFYPREAKVQWKVDNALQSGNSQESVTEQDSKDSTYALFSVLTLSKADYEKHKVYACE<br>VTHQGLSSPVTKSFNRGECDKTHTPPCPAPELLGGPSGGGGSFGMQVPPPAHMGFRSS
ZWB90 H2	QVQLVQSGAEVKKPGASVKVSCKASGYTFTDYYMHWVRQAPGQGLEWMGRVNPNRRGTTYN<br>QKFEGRVTMTTDTSTSTAYMELRSLRSDDTAVYYCARANWLDYWGQGTTVTVSSASTKGPSVF<br>PLAPSSKSTSGGTAILGCLVKDYFPEPVTVSWNSGALTSGVHTSPAVLQSSGLYMLASAVTVPSS<br>SLGTQTYICNVNHKPSNTKVDKKVEPKSCDKTHTPPCPAPELLGGPSGGGGSKAHPWFFGKIPR<br>AKAEEMLNKQRHDGAFLIRESESAPGDFSLSVKFGNDVQHFKVLRDGAGKYFLWVVKFNSLNEL<br>VDYHRTTSVSRNQQIFLRD
ZWB90 L2	DIQMTQSPSSLSASVGDRVTITCSVSSSVSSIYLHWYQQKPGKAPKLLIYSTSNLASGVPSRFSGS<br>GSGTDFTLTISSLQPEDFATYYCQVYSGYPLTFGGGTKVEIKRTVAAPSVAIFPPSDEQLKSGTAS<br>VVCVLNNFYPREAKVQWKVDNALQSGNSQESVTEQDSKDSTYALFSVLTLSKADYEKHKVYACE<br>VTHQGLSSPVTKSFNRGECDKTHTPPCPAPELLGGPSGGGGSKAHPWFFGKIPRAKAEEMLNK<br>QRHDGAFLIRESESAPGDFSLSVKFGNDVQHFKVLRDGAGKYFLWVVKFNSLNELVDYHRTTSV

	SRNQQIFLRD
Emi-kappa VL -GFP11β	DIQMTQSPSSLSASVGDRVTITCSVSSSVSSIYLHWYQQKPGKAPKLLIYSTSNLASGVPSRFSGS GSGTDFTLTISSLQPEDFATYYCQVYSGYPLTFGGGTKVEIKRTVAAPSVAIFPPSDEQLKSGTAS VVCVLNNFYPREAKVQWKVDNALQSGNSQESVTEQDSKDSTYALFSVLTLSKADYEKHKVYACE VTHQGLSSPVTKSFNRGECGGGGSGGGGSRDHMVLHEYVNAAGIT

Supplementary **Movie S1**. For confocal Z-axis stacks, three-dimensional (3D) stereograms showed ZWB90-3 endocytosis in HCC827 cells. Images were acquired at 100X magnification.

Supplementary **Movie S2**. HCC827 cell growth status after treatment with ZWB90-3 was observed after 6 days using the Incucyte® imaging system in real-time. Images were acquired at 20X magnification.

Supplementary **Movie S3**. Under normal conditions, HCC827 cells retained their normal growth state. Images were acquired at 20X magnification.

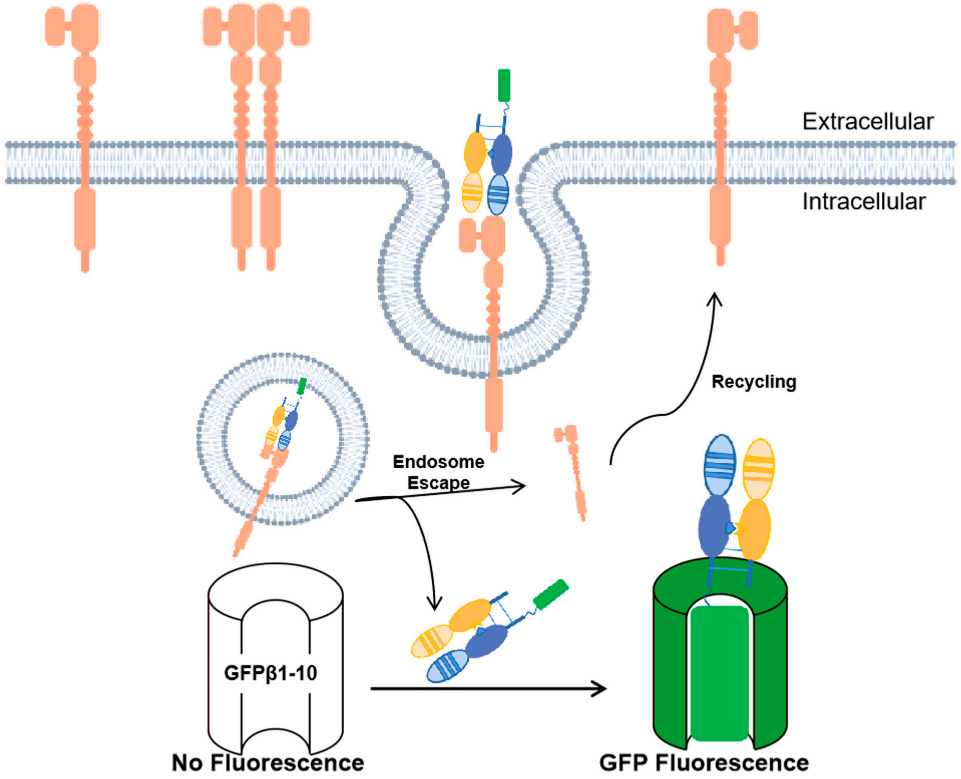

Supplementary **Figure S1**. Representation of the study concept. GFPβ11 peptide fusion proteins were bound to the c-MET molecules on the cell surface and then endocytosed into cells through the antibody-mediated receptor internalization. GFPβ11 peptide fusion proteins escaped into the cytoplasm by an endosomal escaping mechanism. The binding of the GFPβ11 peptide to the non-fluorescent GFPβ1-10 protein fragment in the cytoplasm induced the formation of the active GFP fluorescent chromophore.

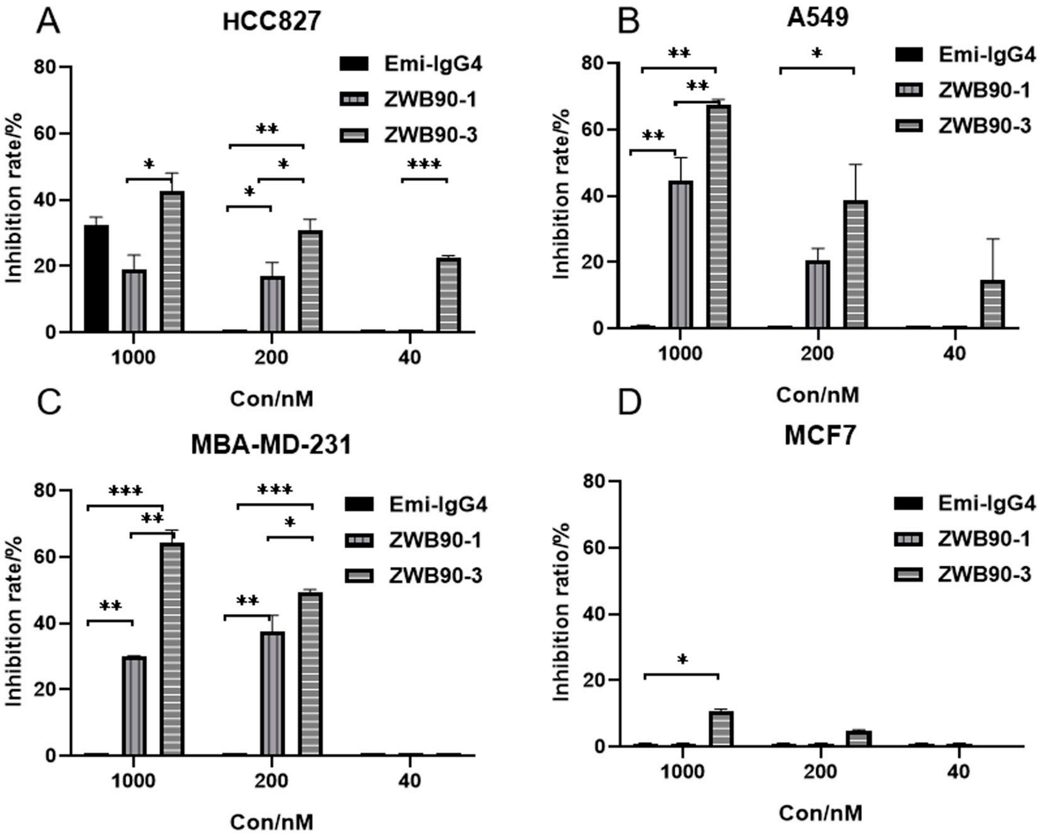

Supplementary **Figure S2**. Effects of ZWB90-1,ZWB90-3 and Emi-IgG4 on the inhibition of cell proliferation in a variety of tumor cells. (**A-D**) HCC827, A549, MBA-MD-231, and MCF7 cells were treated with ZWB90-1, ZWB90-3, or Emi-IgG4 for 100h at 37°C. Thecell inhibition rates (%) were calculated.***P*<0.01 and ****P*<0.001

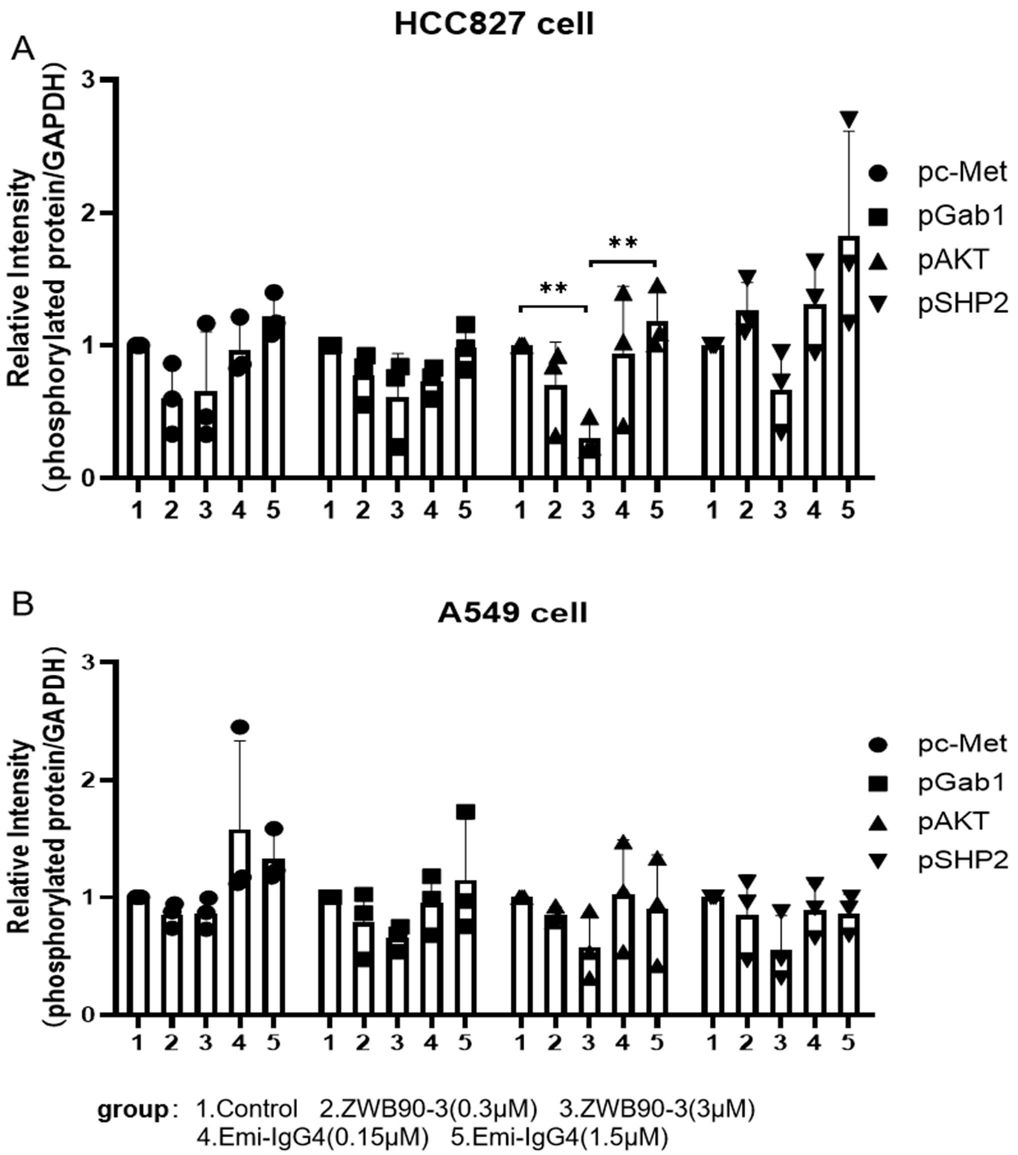

Supplementary **Figure S3**. Relative intensities of phosphorylated proteins were measured and quantified using ImageJ. (**A**) HCC827 cells. (**B**) A549 cells. Experiments were performed in three independent repeats.

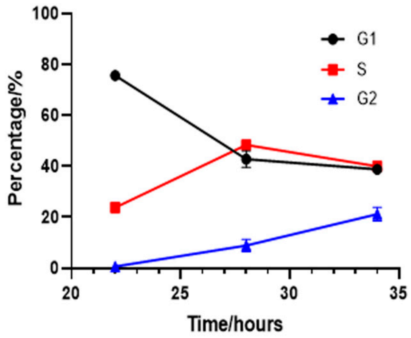

Supplementary **Figure S4**. The time-course curve of ZWB90-3-induced G2/M-phase arrest in A549 cells.

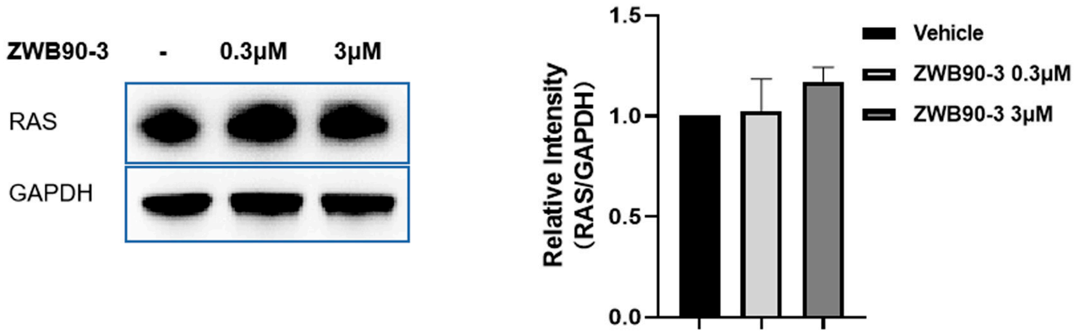

Supplementary **Figure S5**. Western blot analysis was performed to determine the expression level of Ras, where GAPDH served as the loading controls.

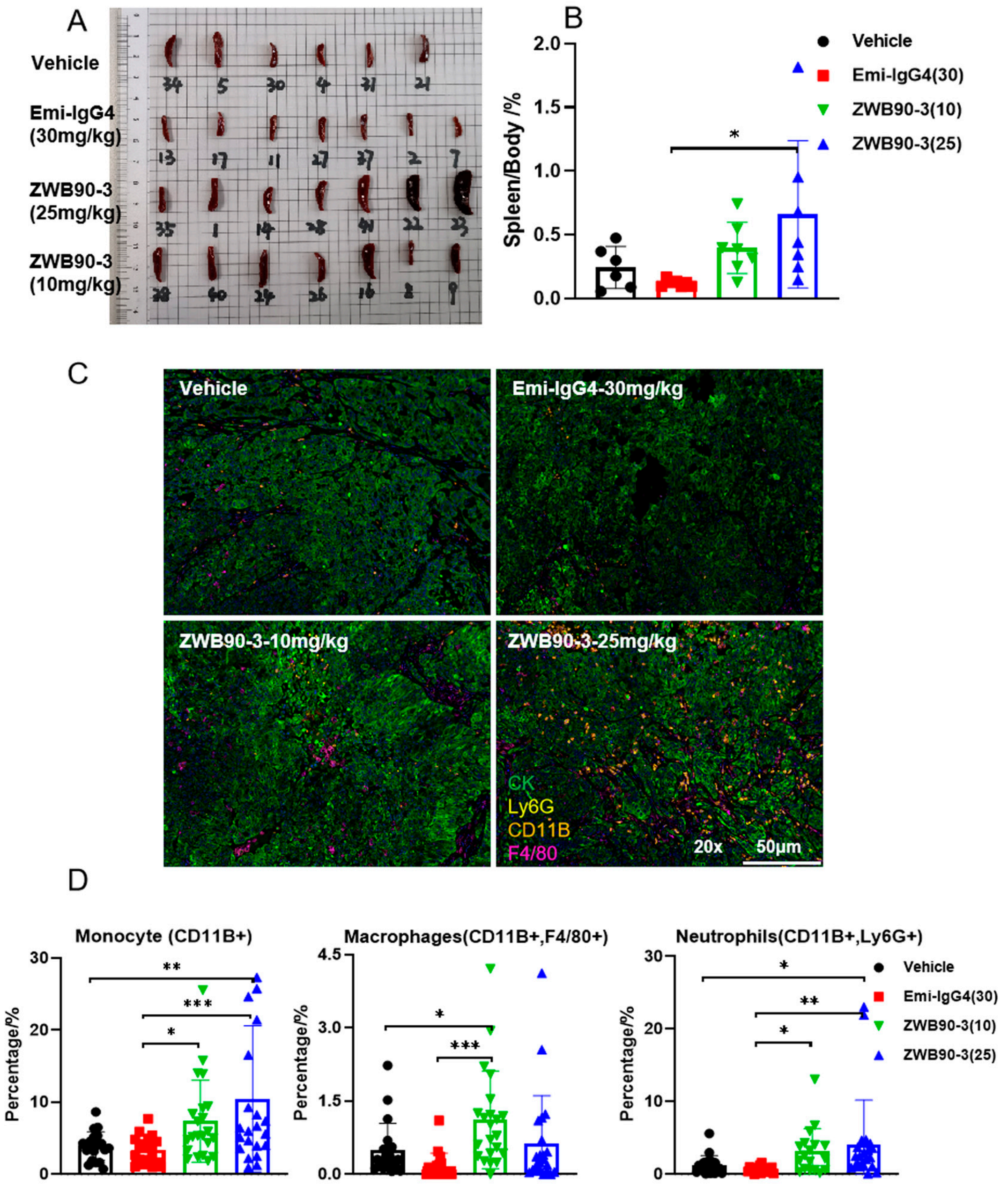

Supplementary **Figure S6**. Immunoreactivity of ZWB90-3 in the A549 xenograft mouse tumor model. (**A**) Spleen tissues of mice were dissected and photographed. (**B**) Spleens were weighed and the spleen-to-body weight ratios were calculated. **P*<0.05 was considered significant compared to the PBS-treated control. All the data are expressed as mean±SD. (**C**)Multiplex IF showed cells were labeled to distinguish monocyte(CD11B+),neutrophils (CD11b+, Ly6G+) and macrophages (CD11b+, F4/80+)on the paraffin-embedded tumor sections.Cytokeratin(CK) was used to stain the A549 tumor cells.(**D**) Quantitative analysis of monocyte,neutrophils and macrophages in the non-necrotic regions of tumor samples to assess the expression levels of inflammatory cytokines. **P*<0.05,***P*<0.01 and ****P*<0.001
